# Differentiation of Atypical Lipomatous Tumors from Lipomas: Our Experience with Visual Analysis of Conventional Magnetic Resonance Imaging

**DOI:** 10.3390/jimaging11020047

**Published:** 2025-02-08

**Authors:** Luz Maria Moran, Chao Yuan Li Cai, Alberto Ramirez, Ana Royuela

**Affiliations:** 1 Department of Radiology, Hospital Universitario Puerta de Hierro Majadahonda, 28222 Madrid, Spain; albert_unit96@hotmail.com; 2 Department of Medicine, Faculty of Medicine, Universidad Autonoma Madrid, 28049 Madrid, Spain; chaoyuanlicai8@gmail.com; 3 Biostatistics Unit, Hospital Universitario Puerta de Hierro Majadahonda, IDIPHISA, CIBERESP, ISCIII, 28222 Madrid, Spain; aroyuela@idiphim.org

**Keywords:** soft tissue tumors, magnetic resonance imaging (MRI), atypical lipomatous tumor (ALT), well differentiated liposarcoma (WDL), lipoma, murine doble minute 2 (MDM2)

## Abstract

Differentiating atypical lipomatous tumors (ALTs) from lipomas using imaging techniques is a challenge, and the biopsy with immunohistochemical determination of murine double minute 2 (MDM2) oncogene is the gold standard. We are looking for a management algorithm with the visual analysis of magnetic resonance images in these two fatty soft tissue tumors that allow us to avoid some biopsies. Two radiologists, blinded to the final diagnosis, independently assessed various features on conventional magnetic resonance imaging (MRI), in 79 patients with pathologically confirmed fatty tumors as either lipoma (MDM2 negative) or ALT (MDM2 positive). Results: The interobserver agreement for the most MRI features was moderate and the musculoskeletal radiologist accuracy for final diagnosis was 90% sensitivity and 66% specificity. Tumors with homogeneous fat signals and a maximum size < 8 cm were always lipomas (*p* < 0.001), and the tumors with septa thickness ≥ 2 mm, or more than one non-fat nodule, and a maximum size ≥ 12.8 cm were typically ALTs. While those tumors with septa < 2 mm or one non-fat nodule, independently of maximum size, the diagnosis of lipoma versus ALT is uncertain and a biopsy is required.

## 1. Introduction

Fatty tumors, particularly lipomas, account for nearly half of soft tissue tumors (STTs) [1,2]. The lipoma is a benign mesenchymal tumor composed of slow-growing adipose tissue, typically located between the skin and the muscle layer. In contrast, liposarcomas are malignant tumors of mesodermal origin, also derived from adipose tissue, and are usually found beneath the deep fascia [3]. Among the liposarcomas, well-differentiated liposarcomas (WDL) are the least malignant. Depending on their anatomical location, they are referred to as atypical lipomatous tumors (ALTs) when arising in the extremities, chest wall, or abdomen wall. However, when located intrathoracically or intraabdominally, the term well-differentiated liposarcoma (WDL) is maintained [4]. From a histopathological perspective, ALT and WDL are synonymous, sharing identical morphology, karyotype, and biological behavior [5].

Biopsy with immunohistochemical analysis of murine double minute 2 (MDM2) oncogene amplification is the gold standard. This oncogene is expressed in ATL, but not in lipomas [6]. Differentiating between lipoma and ATL/WDL using imaging techniques is a significant challenge, even for highly experienced radiologists. However, this differentiation is crucial, as lipomas generally do not require treatment unless they become large and compress nearby structures, whereas ATL/WDL must be treated surgically due to their potential to dedifferentiate into liposarcoma with metastatic potential [7,8,9]. Furthermore, in cases of ATL/WDL, surgery should aim wide, with tumor-free margins, while for lipoma, marginal excision is sufficient if surgery is indicated [10]. Additionally, patients with ATL require long-term clinical follow-up, as late dedifferentiation may occur, even 5–10 years after resection or recurrence [5,11].

MRI is the standard imaging modality for the assessment of soft tissue tumors, including fatty tumors. Various studies have analyzed the MRI characteristics of these tumors [12,13,14,15,16]. Kransdorf et al. found that a tumor length greater than 10 cm, the presence of thick septa, and non-fatty nodular areas are associated with ALT [12]. Doyle demonstrated that the absence of septa or non-fatty nodules, along with homogeneous signal suppression on selectively fat-suppressed sequences, was predominantly associated with lipomas rather than ALT [13]. A multicenter study by Nardo et al. revealed that qualitative MRI variables can assist in differentiating lipomas from ALT, although the contrast enhancement may sometimes lead to confusion [14]. However, there is an overlap in these MRI features used to distinguish ALT from lipoma. These studies have shown that MRI features can help differentiate lipomas from ALTs, but all of these features had not proven to be definitive.

In our study, we evaluated the accuracy of each MRI feature independently to differentiate between these two deep fatty soft tissue tumors, and we implemented other MRI features, such as the number and the size of solid non-fatty nodules. Based on the analysis of these MRI features, we are looking for a management algorithm that allows us to reduce the number of biopsies with MDM2 determination to differentiate these two deep fatty soft tissue tumors.

## 2. Materials and Methods

Retrospective observational study approved by the Research Ethics Committee of the hospital under file PI-41/23.

### 2.1. Study Population

Consecutive magnetic resonance imaging studies conducted between 2018 and 2022 in patients with pathologically confirmed lipomas and ALT, were retrieved from the PACS. The inclusion criteria were all subjects had underwent a MRI study with protocol that included T1W, T2W, and PD-SPAIR sequences, and a biopsy with MDM2 oncogen determination within 1–2 months after MRI.

Exclusion criteria included the absence of prebiopsy MRI, suboptimal MRI quality, and superficial lipomatous tumors (those located in the skin and subcutaneous tissue). Additionally, fatty tumors clearly identifiable as intramuscular lipomas with intralesional muscle fibers were excluded from the MRI study. All soft tissue fatty tumors collected in this study were deep, located below the deep fascia.

### 2.2. Clinical Data Collection

Clinical data were retrieved from the Electronic Health Record Systems without changing data or directly contacting any patient. The following clinical variables were assessed: age, sex, and anatomical location of the tumors within the body (upper limb: arm and forearm; superficial trunk: neck, chest wall, and abdominal-pelvic wall; and lower limb: thigh and leg).

### 2.3. MRI Protocol

Magnetic resonance imaging studies were performed on either 1.5 or 3.0 Teslas magnetic field strength in different scanners, including Achieva Nova, Philips Healthcare, Netherlands and Magneton Sola and Magneton Vida, Siemens Healthineers, Germany. The antennas were adjusted to the anatomic area and to the size of the tumor under study. The conventional MRI protocol consisted of a set of a T1-weighted spin-echo sequence (SE T1), a T2-weighted turbo spin-echo sequence (TSE T2), and Proton Density-Spectral Attenuated Inversion Recovery (PD-SPAIR) imaging for all patients. The sequences included axial SE T1, TSE T2 and PD-SPAIR, and the long axis of the tumor (coronal or sagittal SE T1).

### 2.4. Evaluation of the MR Images

MR images were independently evaluated by two radiologists, one specialized in musculoskeletal radiology with over 15 years of experience (radiologist 1), and a general radiologist. Their assessments were used to measure interobserver agreement for each MRI feature. The cases were anonymized and presented in random order, without access to clinical or histological data.

The following conventional MRI parameters were analyzed, excluding contrast enhancement and based on previous studies: (1) signal characteristics (fatty homogeneity versus heterogeneity in T1, T2 and DP-SPAIR), (2) presence of septa, defined as non-fat linear tissue within the mass, (3) thickness of the thickest septum in millimeters (mm) (<2 mm or ≥2 mm), (4) presence of non-fat nodules, (5) number of non-fat nodules (one or more than one), (6) size of the largest nodule (<10 mm or ≥10 mm), (7) tumor size (maximum diameter in centimeters, cm), and (8) tumor margins (well-defined or poorly defined).

### 2.5. Statistical Analysis

Qualitative variables were described using absolute and relative frequencies, while quantitative variables were reported as mean and standard deviation (SD) or median and interquartile range (25th and 75th percentiles; p25, p75). Univariate analyses were performed using Pearson chi-square test or Fisher’s exact test, as appropriate. Interobserver agreement was assessed using Cohen’s kappa index, following the interpretation suggested by Landis and Koch [15], with a kappa < 0 being a poor agreement; between 0.01 and 0.2 slight agreement; 0.21–0.40 fair agreement; 0.41–0.60 moderate agreement; 0.61–0.80 substantial agreement, and 0.81–1.00 almost perfect agreement. For variables with more than two ordered categories, quadratic weights were applied to estimate the kappa index. In cases where the outcome’s prevalence was very low, the kappa index was penalized, and the Brennan and Prediger coefficient was used instead.

The median and 25th and 75th percentiles were used to describe differences between the two diagnoses for numerical variables, with differences assessed using the Mann–Whitney U test. The area under the ROC curve (AUC) was used to evaluate the discriminatory ability of the variables and an optimal cutoff point was considered when the AUC was greater than 0.7.

The optimal cut-off point was estimated using the Liu method.

Diagnostic accuracy for the musculoskeletal radiologist was evaluated by estimating sensitivity, specificity, and likelihood ratios (positive and negative).

Statistical analysis was performed using the Stata program version 18 (StataCorp. 2023. Stata Statistical Software: Release 18. College Station, TX, USA: StataCorp LLC).

## 3. Results

### 3.1. Patient Characteristics and Tumor Localization

A total of 79 patients with deep fatty soft tissue tumors were studied in our hospital, with immunohistochemical determination of MDM2 for the diagnosis of ALT.

Among these patients, there were 59 lipomas, with 29 (49%) in women and 30 (51%) in men, and a mean age of 60.6 years (SD 9.8). There were 20 cases of ALT, with 8 (40%) in women and 12 (60%) in men, and a mean age of 62 years (SD 11.1).

In terms of location, most fatty tumors were in the lower limb (*n* = 38) (48%), and were almost equally distributed between lipomas and ALT (with 19 in each group) This was followed by the superficial trunk with 18 tumors, where lipomas predominated (94.4%). In the upper limbs, all the 23 tumors were lipomas (see Table 1).

Among the ALT cases, sixteen (80%) were in the thigh, three in the leg, and one in the superficial trunk, specifically in the thoracic wall. The lipomas were distributed practically almost equally across the upper and lower limbs as well as the superficial trunk.

### 3.2. MR Imaging Features

The results of the MRI variables, along with interobserver agreement, are outlined in Table 2.

**Figure 1:**
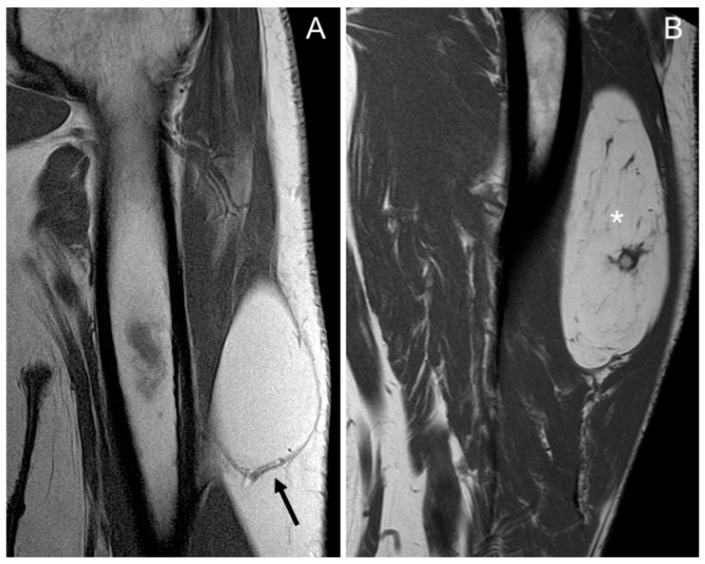
Coronal T1 weighted (T1W) images: (**A**) homogeneous lipomatous (completely fatty) tumor in the left arm (arrow), and (**B**) heterogeneous lipomatous mass in the left thigh, with one non-fat nodule (*) and multiple septa. The diagnosis for both tumors is lipoma, confirmed as MDM2 negative.

In total, 97–100% of tumors with homogeneous fat signal in the three sequences (*p* value < 0.001) were lipomas, and 55–61% of lesions with heterogeneous signal were lipomas (Figure 1).

**Figure 2:**
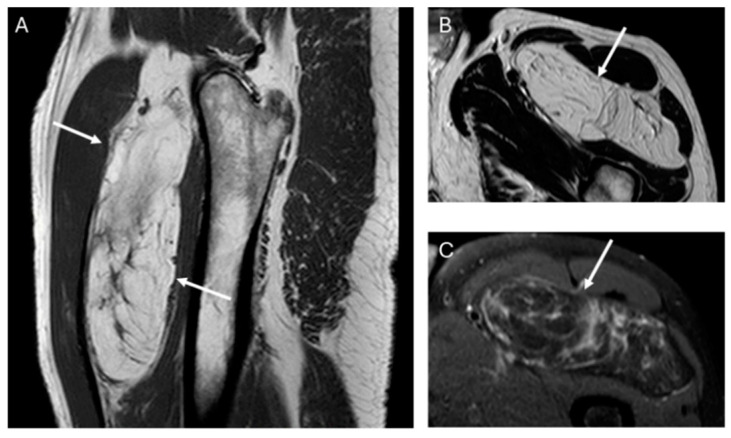
Sagittal T1W (**A**) and axial T2W (**B**) and DP SPAIR (**C**) MR images with a heterogeneous lipomatous mass (arrows) in the anterior compartment of the left thigh. The final diagnosis was atypical lipomatous tumor (ALT) confirmed as MDM2 positive.

Fifty-four of the 59 lipomas had not septa or septa < 2 mm, while nineteen of 20 ALT had septa and these were ≥2 mm in 38%, (*p* value = 0.001) (Figure 2).

The presence of non-fat nodules was not significant, as there were no differences between lipomas and ALT (*p* value = 0.096). Twenty lipomas and ALTs presented non-fat nodules, of which one out of twelve lipomas and six out of eight ALTs showed more than one non-fat nodule (*p* value < 0.001). However, there were no significant differences in the size of the largest non-fat nodule between lipomas and ALTs (*p* value = 0.550).

Forty-nine of fifty-seven tumors (86%) with well-defined margins were lipomas (*p* value < 0.001).

Regarding tumor size, considering the long axis of the tumor, the median size was 9.7 cm (p25; p75: 7; 12 cm) for lipomas, and 15 cm (12; 17) for ALTs, with significant differences between both tumors (*p* value < 0.001). The AUC ROC for differentiating between lipomas and ALTs was 0.847. The optimal cut-off point obtained from the AUC, using the Liu method, was 12.8 cm. We also pursued another cut-off point of 8 cm, which maximized the sensitivity.

The interobserver agreement between the musculoskeletal and general radiologists was moderate for most considered variables, substantial for defining margins, and almost perfect for the feature of tumor size using the 8 cm cut-off point (Table 2).

The diagnostic accuracy of the senior musculoskeletal radiologist vs. compared to the gold standard MDM2 gene test showed 90% sensitivity (95% CI: 68 to 99) and 66% specificity (95% CI: 52 to 78). The positive likelihood ratio was 2.61 (95% CI: 1.78 to 3.83) and the negative likelihood ratio was 0.15 (95% CI: 0.04 to 0.58).

**Figure 3:**
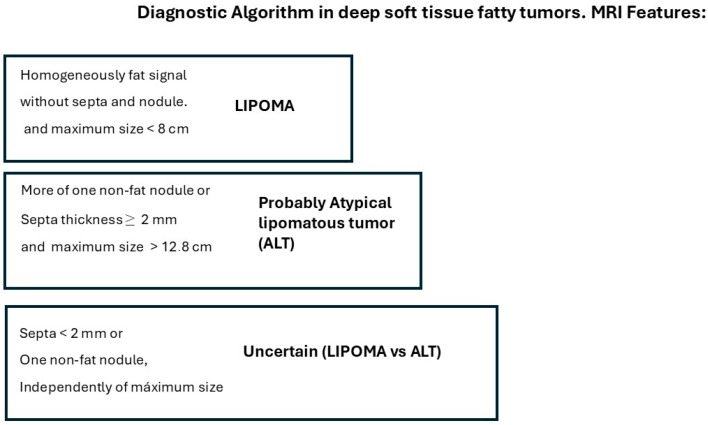
MRI algorithm proposed for the management of deep fatty soft tissue tumors in extremities and superficial trunk.

We propose the following approach for managing deep fatty soft tissue tumors based on their MRI characteristics, as illustrated in Figure 3. When a fatty tumor demonstrates homogeneous fat signal intensity, lacks septa and non-fat nodules, and has a maximum dimension of less than 8 cm, it is most likely a lipoma. Conversely, when a deep fatty tumor exhibits septa ≥ 2 mm, more than one non-fat nodule, and a maximum dimension greater than 12.8 cm, it is most likely an ALT. Additionally, if MRI reveals a fatty soft tissue tumor with heterogeneous signal intensity, septa < 2 mm, or a single non-fat nodule—regardless of the tumor’s maximum size—a biopsy with MDM2 determination is required to characterize the tumor before initiating treatment.

## 4. Discussion

According to Kransdorf, when a tumor consists entirely of fat signal intensity and any fibrous septa present is thin, a diagnosis of lipoma can be made. Features that suggest ALT/WDL in a fatty tumor include older patient age, the presence of thick septa (≥2 mm), large tumor size (>10 cm), reduced fat content, and nodular-globular areas of non-fat tissue within the tumor [12]. Other authors, like Gaskin and O’Donnell, have reported that a deep location beneath the fascia around muscles and tumor size > 5 cm are more common in ALT than in lipomas [5,10].

**Figure 4:**
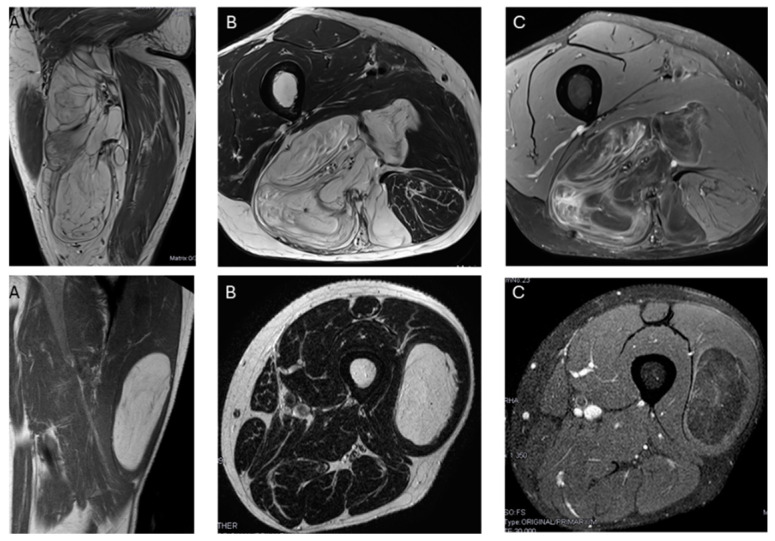
**Top**: Heterogeneous lipomatous mass in the posterior compartment of the thigh, measuring 22 cm, with multiple thick septa visible on T1 and T2 (**A**,**B**) and showing incomplete suppression on DP SPAIR (**C**). These MR findings are consistent with a radiological diagnosis of ALT. Final diagnosis: ALT (MDM2 positive). **Bottom**: Lipomatous tumor in the left lateral vastus, measuring 14 cm, with thin septa visible on T1 and T2 (**A**,**B**) and incomplete suppression on DP-SPAIR (**C**), leading to a radiological diagnosis of uncertain lipomatous tumor (lipoma versus ALT). Final diagnosis: lipoma (MDM2 negative).

In our series, the lipomas showed both homogeneous and heterogenous fat signal intensity, while all ALTs were heterogenous tumors. Based on this criterion—the signal intensity of the tumor on MRI—some lipomas may be misinterpreted as ALT, but the opposite is not true; no ALTs would be misdiagnosed as a lipoma. With homogeneous hyperintensity on SE T1 and TSE T2 sequences, and complete suppression of the fat signal using fat suppression techniques, the diagnosis of lipoma can be made with high confidence. In contrast, heterogeneous tumors may be either a lipoma or an ALT (Figure 4) Therefore, homogeneous signal rules out ALT, while heterogeneous signal does not rule out lipoma (Figure 1).

**Figure 5:**
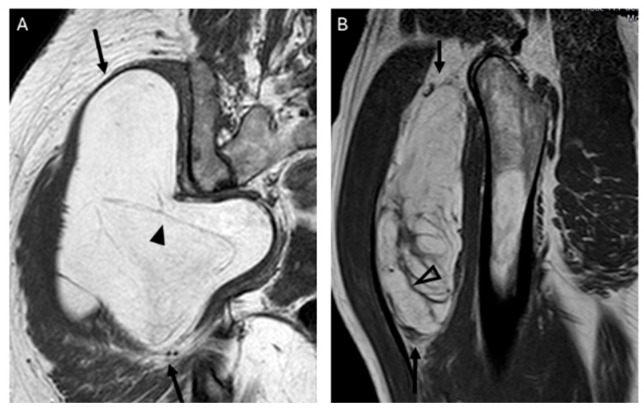
Septations. Coronal T1W image (**A**) showing a lipomatous tumor (black arrows) with thin septa < 2 mm (arrowhead) in the gluteal region. Final diagnosis: lipoma (MDM2 negative). Sagittal T1W image (**B**) showing a lipomatous tumor (black arrows) with thick septa ≥ 2 mm (open arrowhead). Final diagnosis: atypical lipomatous tumor (MDM2 positive).

The presence and thickness of septa show significant differences between lipomas and ALTs. This one agrees with the findings of Kransdorf and Hosono, where the septa were thick and irregular in ALT/WDL and thin in lipoma [12,16]. Similarly, Brisson observed that lipomas were isointense to subcutaneous fat and may contain a few thin septa [17]. In our series, the absence of septa indicates lipoma and septa ≥ 2 mm is suggested of ALT. Septa < 2 mm can be indicative of either lipoma or ALTs (Figure 5).

Regarding the presence and size of non-fat nodules, there was no difference between lipomas and ALTs; however, in our series, there was a difference in the number of nodules.

In lipomas, these non-fat nodules correspond to fat necrosis, calcification, fibrosis, inflammation and myxoid change [12], because the mature fat is subject to various superimposed secondary inflammatory processes. In ALT, non-fat nodules are also common, although at least 75% of tumor is composed of fat.

**Figure 6:**
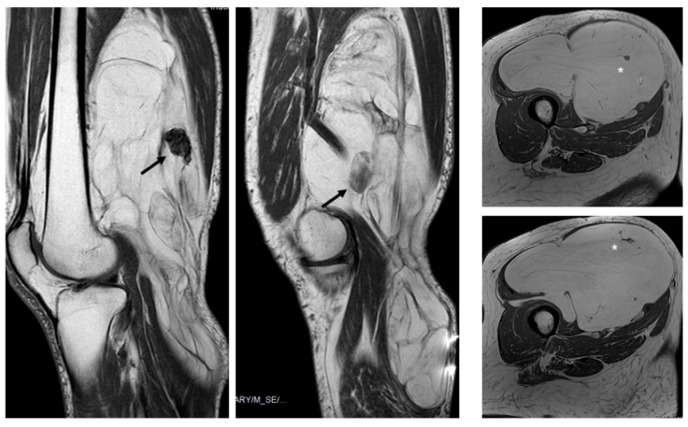
Nodules. Sagittal T1W images show a deep lipomatous mass in the popliteal fossa, measuring 27 cm along its long axis and with two non-fat nodules (arrows) Diagnosis: ALT (MDM2 positive). Axial T2W images present a lipomatous tumor in quadriceps, with two non-fat nodules (asterisks) Diagnosis: ALT (MDM2 positive).

There was typically more than one non-fat nodule in ALT, while lipomas usually had only one (Figure 6). Unlike other studies, which suggested that non-fat nodules larger than 1 cm in diameter are an important discriminator favoring ALT over lipoma [17], our findings did not support this distinction.

We do not know the exact explanation for these results, but ALTs are generally larger than lipomas, which may explain why they tend to have more than one non-fat nodule, although these nodules are not significantly larger in ALTs than in lipomas.

Kransdorf’s series indicated that 31% of lipomas presented non-fatty areas [12]. These non-fat areas contained necrosis or inflammation within the tumor, but showed no cellular atypia, resulting in similar appearance to ALT. This similarity explains why some lipomas may have been over-diagnosed as ALT/WDL based on MRI features.

Lipomas exhibited well-defined margins in 83% of cases, which showed significant differences compared to ALTs, which predominantly had poorly defined and infiltrative margins. The average maximum dimension of ALTs was greater than that of lipoma, which is consistent with many previous studies. In Kransdorf’s work, a long axis greater than 10 cm was associated with ALT [12], while Knebel et al. found that a long axis greater than 13.0 cm indicated ALT [6]. We selected a cutoff point of 8.0 cm for the long axis of the tumor to ensure that no ALT goes undiagnosed.

The visual analysis of the radiologist showed better sensitivity than specificity by using these imaging criteria, leading to an overdiagnosis of ALT on MRI, while histology confirmed lipoma. This overdiagnosis explains some false positive cases in imaging diagnosis. This may be due to the imaging appearances of certain lipomas that resemble ALT when we are employing strict MRI criteria, such as a completely fatty tumor without septa or non-fat nodules.

Regarding tumor location, the lower limb was identified as a significant predictor of ALT, while the upper limb was associated with lipomas. In our study, thighs were the most prevalent location for both lipomas and ALT, consistent with findings from other studies [6,12,18,19].

The epidemiological variables, patient age, and sex did not differentiate between lipoma and ALT. The average age of onset for both tumors was in the seventh decade of life. These results are consistent with some works [13,19,20], but differ from others [6,12,17], where ALT patients were significantly older than lipoma patients. None of the studies showed statistically significant differences in gender between lipomas and ALTs.

There are some limitations in this study. First, septa and non-fat nodular area enhancement were not investigated. Although Nardo’s multicenter study concludes that qualitative MR variables help in characterizing lipomas and ALTs, contrast enhancement does not, and may even cause confusion [14]. Additionally, contrast-enhanced MRI sequences increase both the time and cost of the procedure. Another limitation is the size imbalance between the lipoma and ALT samples, which reflects what happens in clinical practice. The differences in sample size may affect the results, aside from the bias introduced by the subjective visual analysis of MRI images by radiologists who tend to overdiagnosis ALT. Our approach to managing deep fatty soft tissue tumors based on their MRI characteristics, (Figure 6) would reduce the number of biopsies required for these two tumor types, minimize uncertainty for expert radiologists, and provide significant support to less experienced or generalist radiologists without specific training in soft tissue tumors.

We intend to conduct a prospective study on a sample of deep fatty tumors evaluated with MRI and assessed by general radiologists using our algorithm to determine radiologic accuracy compared to the gold standard (MDM2 determination).

At the same time, and given the complexity of characterizing these tumors based on imaging alone, our team is also beginning to implement artificial intelligence. We are applying various segmentation algorithms, calculating radiomic variables, and using supervised machine learning models with the goal of developing classifiers for these two tumor types. Preliminary results are promising, particularly in improving specificity.

In conclusion, the proposed algorithm for MRI image analysis assists less experienced radiologists in evaluating the images, and generally provides confidence when avoiding diagnostic biopsy. Meanwhile, artificial intelligence emerges as a useful tool to aid in the characterization of these tumors.

## Figures and Tables

**Table 1:** Tumor location and distribution based on lipoma/ALT diagnosis.

	Lipoma	ALT	*p*-Value
Upper limbs	23 (39%)	0 (0%)	<0.001
Superficial trunk	17 (29%)	1 (5%)	
Lower limbs	19 (32%)	19 (95%)	

**Table 2:** Results of MRI variables for the musculoskeletal radiologist and interobserver agreement (kappa).

	Lipoma (*n* = 59)	ALT (*n* = 20)	*p*-Value	Kappa Index (95% CI)
**T1 Homogeneity**				
Homogeneus	27 (100%)	0 (0%)	<0.001	0.536 (0.348; 0.724)
Heterogeneus	32 (61.5%)	20 (38.5%)		
**T2 Homogeneity**				
Homogeneus	29 (100%)	0 (0%)	<0.001	0.544 (0.349; 0.739)
Heterogeneus	30 (60%)	20 (40%)		
**DP SPAIR Homogeneity**				
Homogeneus	36 (97.3%)	1 (2.7%)	<0.001	0.532 (0.338; 0.726)
Heterogeneus	23 (54.7%)	19 (45.3%)		
**Septa (thickness)**				
No septa	27 (96.4%)	1 (3.6%)	0.001	0.531 * (0.378; 0.684)
<2 mm *	27 (69.2%)	12 (30.8%)		
≥2 mm	5 (41.7%)	7 (58.3%)		
**Non-fat nodules**				
Absence	45 (78.9%)	12 (21.1%)	0.096	0.538 * (0.346; 0.730)
Presence	12 (60.0%)	8 (40.0%)		
**Non-fat nodule number (*n* = 20)**				
One	11 (84.6%)	2 (15.4%)	0.001	0.601 * (0.396; 0.806)
More of One	1 (14.3%)	6 (85.7%)		
**Largest Non-fat nodule size (*n* = 20)**				
<1 cm	9 (64.3%)	5 (35.7%)	0.550	0.600 * (0.0; 1.00)
≥1 cm	3 (50%)	3 (50%)		
**Margins**				
Well defined	49 (85.9%)	8 (14.1%)	<0.001	0.669 * (0.537; 0.800)
Poorly defined	10 (45.5%)	12 (54.5%)		
**Tumor size (cm)**				
<8 cm	26 (100%)	0 (0%)	<0.001	0.829 (0.695; 0.963)
≥8 cm	33 (62.3%)	20 (37.7%)		
**Tumor size (cm)**				
<12.8 cm	46 (90.2%)	5 (9.8%)	<0.001	0.790 (0.652; 0.930)
≥12.8 cm	13 (46.4%)	15 (53.6%)		

* Brennan and Prediger index.

## Data Availability

The data are available under reasonable request.
